# Unified Open Hardware Platform for Digital X-Ray Devices; its Conceptual Model and First Implementation

**DOI:** 10.1109/JTEHM.2020.3000011

**Published:** 2020-06-04

**Authors:** F. Aytac Durmaz, Altay Brusan, Cengizhan Ozturk

**Affiliations:** 1 Institute of Biomedical Engineering, Boğaziçi University34684IstanbulTurkey; 2 Durmaz Technology AS34684IstanbulTurkey; 3 Pievision AS34684IstanbulTurkey; 4 Center of Life Sciences and TechnologiesBoğaziçi University34684IstanbulTurkey

**Keywords:** Medical imaging, open source hardware, plug-integrate-play (PIP) medical device development, x-ray applications, x-ray imaging hardware

## Abstract

Background: Digital radiography devices are still the gold standard for diagnosis or therapy guidance in medicine. Despite the similarities between all direct digital x-ray systems, researchers and new companies face significant challenges during the development phase of innovative x-ray devices; each component is manufactured independently, guidance towards device integration from manufacturers is limited, global standards for device integration is lacking. Method: In scope of this study a plug-integrate-play (PIP) conceptual model for x-ray imaging system is introduced and implemented as an open hardware platform, SyncBox. The researchers are free to select each individual device component from different vendors based on their intended application and target performance are utilized in criteria. Result: As its first implementation, SyncBox and its platform a full body high resolution radiographic scanner that employs a novel TDI digital detector. Conclusion: We believe that SyncBox has a potential for introducing an open source hardware platform to x-ray equipment design.

## Graphical abstract

We introduce a plug-integrate-play (PIP) conceptual model for an x-ray imaging system and implement it as an open hardware platform, SyncBox. Based on validation studies, we believe that SyncBox holds potential for introducing an open source hardware platform to x-ray equipment design.

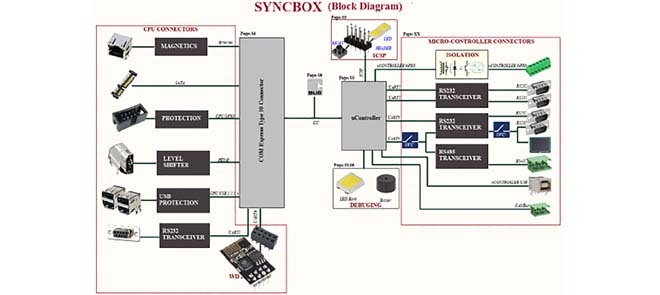

## I. Introduction

There are common underlying design principles and components in x-ray based projectional imaging modalities; such as chest radiography, linear and multi-directional tomography, mammography, bone density and skeletal radiography devices.

**FIGURE 1.**
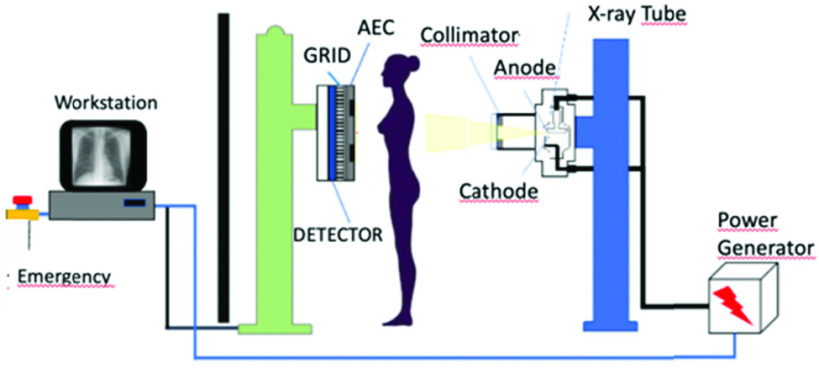
A general schematic of a typical radiography scanner. High frequency generator produces the energy pulse and sends it through the x-ray tube. A collimator targets the x-ray beam over the target. Photons past through the subject and acquired by a detector. Digital image is transmitted to a dedicated workstation from the detector.

A standard x-ray device (Fig. 1) includes a high frequency generator connected to a x-ray tube, a gantry area, a command console for exposure control and a detector for image acquisition [1]. In addition to these, there are several additional components frequently present in a medical x-ray imaging system: a motorized, fixed or mechanical collimator is used to navigate and narrow the beam of x-ray onto a target area; automatic exposure control (AEC) system to compensate the variations of the x-ray exposure towards the patient and maintain the image quality depending on the radiation level coming through the detector, Dose Area Product (DAP) system to measure the radiation level exposed to the patient and a workstation to digitally control monitor and acquire the x-ray system [1]–[6]. Furthermore, x-ray scanners employ various additional modules; there are collision and safety sensors, cooling systems, grids, electro-mechanical and robotic instruments, security & interlock mechanisms and networking and storage capabilities [2], [3], [6], [8]. Integrating all the above-mentioned components is needed to a complete a radiography scanner.

Device vendors make a new radiography scanner by selecting the off-the-shelf components from different manufacturers based on the final device workload, budget and quality criteria and then introduce the combined system to the market. However, there is no accepted standard for component connectivity and communication, therefore the device integration process takes a considerable amount of time and effort. This process often fails to eliminate all the uncertainties of the design; safety and efficiency issues are encountered during the clinical usage of these devices [9]. Imaging devices require a efficiency, safety and regulatory review whenever a component is replaced. Extensive device testing and calibration should be performed again costing significant time and money for each finite improvement.

On the other hand, researchers, who are interested in developing image processing and decision-making algorithms, face inaccessible systems. In order to avoid risks, vendors restrict user access levels and hardware intervention capabilities. This conservative attitude limits the researchers to pre-configured options therefore making advanced research on these modern medical imaging devices very challenging.

Complementing open platform-based trends in medical device development, we introduce here a conceptual model for digital x-ray scanners and an open source hardware device and platform, named ‘SyncBox’. This platform employs Plug-Integrate-Play (PIP) manufacturing paradigm to the x-ray radiography device design and introduces a potential solution for its integrability and extensibility problems. SyncBox theoretically could implement all x-ray devices working with direct imaging principle such as DR systems, mammography, fluoroscopy, mobile x-ray devices, and angiography. It has advantages for shorten the research and development process for a new x-ray device integration, and offers a significant performance and quality comparing to a self-developed x-ray control systems.

In the following parts of this paper, the background of Plug and Play (PnP) in medicine is discussed next. Methodology Section discusses the x-ray device model and introduces the SyncBox concept in detail. Results Section describes the first implementation of this platform to develop a special time-delay integration scanner. Discussion section evaluate the initial results, its current limitations and how these systems could be improved. Potential impact of this device on medical industry discussed in the Conclusion Section.

## II. Background

Healthcare delivery organizations are starting to view the interoperability gap as a real problem: a barrier to innovations that could potentially improve patient safety and health care affordability [10]. Medical device interoperability is the ability of medical devices, clinical systems, or their components to communicate with each other in order to safely fulfill an intended purpose [11]. Effective interoperable medical systems should be safe, secure and usable at all levels of conjunction and require holistic view [12]. Tolk *et al.* postulated a model consisting of five levels for conceptual interoperability [13], which was then extended to seven levels by Turnitsa *et al.* [14]. These models characterize the internal and external interactions occurring in systems. Robkin *et al.* [15] extended these models for medical devices and health care systems.

The standardization bodies, consortium of manufacturers and academic researchers have been leading an extensive effort to make medical systems interoperable and to create a complete Plug and Play (PnP) system structure [16], [17]. The “Medical Device Plug-and-Play Interoperability program” was founded in the US [17] to develop an open-source clinical environment (OpenICE) [18]. The MD PnP Lab [17] was opened in May 2006 to provide an environment to support projects, testing and prototyping of a vendor-neutral “sandbox” solution [17]. In Germany flagship project OR.NET [19] focused on secure and dynamic interlinkage of medical devices in the operating room and hospital.

Regulatory bodies also have made great contributions on defining the standards. As a context in ISO/IEEE 11073 “Health Informatics - Medical / Health Device Communication Standards”, a set of standards was designed for enabling communication between medical, health care and wellness devices and with external computers [20]. Moreover, the ASTM F2671 “Essential Safety Requirements for Equipment Comprising the Patient-Centric Integrated Clinical Environment (ICE)” [21], is a series of standards that conceptualize excellence in biomedical and health system design and development practices [22], [23].

Association for the Advancement of Medical Instrumentation (AAMI) adopted an interoperability hierarchy model based on Turnitsa model [24] (Table 1). In this model, Level 0 interoperability describes a situation in which two systems have no need to, or cannot, interoperate and the interoperability is accomplished by hand. Technical interoperability (Level 1) is achieved when two systems have the means to communicate but neither has a shared understanding of the structure nor the meaning of the data being communicated. In this level, a stream of bits or bytes could be sent between the systems, however, none of the sides has the facility to interpret the data. Syntactic interoperability (Level 2) occurs when information is communicated with structure but without any meaning. In other words, in this level, both sides know the data format but received data makes no sense. Semantic interoperability (Level 3) is achieved when the data have meaning, but a full understanding of the relationships between elements of the data and the context of the data is missing. Pragmatic interoperability (Level 4) encompasses a shared understanding of the data, the relationships between elements of the data and the context of the data. However, pragmatic interoperability cannot accommodate changing relationships or context. Dynamic interoperability (Level 5) is more flexible, allowing for changing contexts and relationships over time or within the scope of specific transactions [24].

**TABLE 1:** Levels of Interoperability: Different Levels of Interoperability Abilities From 0 (no Interoperability) to 5 (Dynamic Interoperability) [24]

	Level	Name	Description	Standard
Inter-operability	5	Dynamic	Components internal states and capabilities understood	
	4	Pragmatic	Context understood	IHE PCD Continua
Integrability	3	Semantic	Meaning understood	SNOMED Continua / 11073
	2	Syntactic	Common format	Hl7 11073 series
Connectivity	1	Technical	Common physical and transport	Ethernet, Wi-Fi, USB
	0	None	None	

In order to solidify the foundation for a safe, secure and reliable device interoperability, device models have been used. A device model consists of the device context and dynamics. Main purpose of the device model is to support the system designer to build up a safe device design for both machine operator and patients [9], [25]. A device model is usually prepared from different views (e.g. data, dynamics, run-time) and depends on the level of interoperability presented in Table 1; it may contain details such as data encoding format (e.g. float type format), units of measurements (e.g. mmHg, beats/min) and measured parameter (e.g. blood pressure, O2 concentration). ASTM F2761-Medical Devices and Medical Systems Essential Safety Requirements for Equipment Comprising the Patient-Centric Integrated Clinical Environment (ICE) proposes the following principles to increase the safety and security of connections: a The connected equipment does not fail due to receipt of messages or other information. b Failures caused by direct or indirect connection to an interoperable component, electrical and logical mismatching, erroneous commands, receiving and processing erroneous data or commands, or not adhering to the non-functional requirements of the communication specification, should be considered in verification of the system [21].

The mature form of interoperability is Plug-and-Play (PnP). At this level, devices interoperate with each other seamlessly and solely based on configuration without significant engineering effort [13]–[15]. PnP capability could bring great reductions to the burden of patients, physicians and hospitals and it could prevent technical issues and clinical workflow inefficiencies by integrating the data and functionalities of medical devices and clinical information systems [10]. However, a complete PnP implementation is difficult and it requires a multi-layer device model. Device model includes a service model, data format, process, communication protocols depending on the standards [20], [26] control and management properties [15], [27].

In the next section a device model for digital x-ray scanner is introduced. This model is then applied for developing a unified open source hardware platform.

## III. Methodology

**FIGURE 2.**
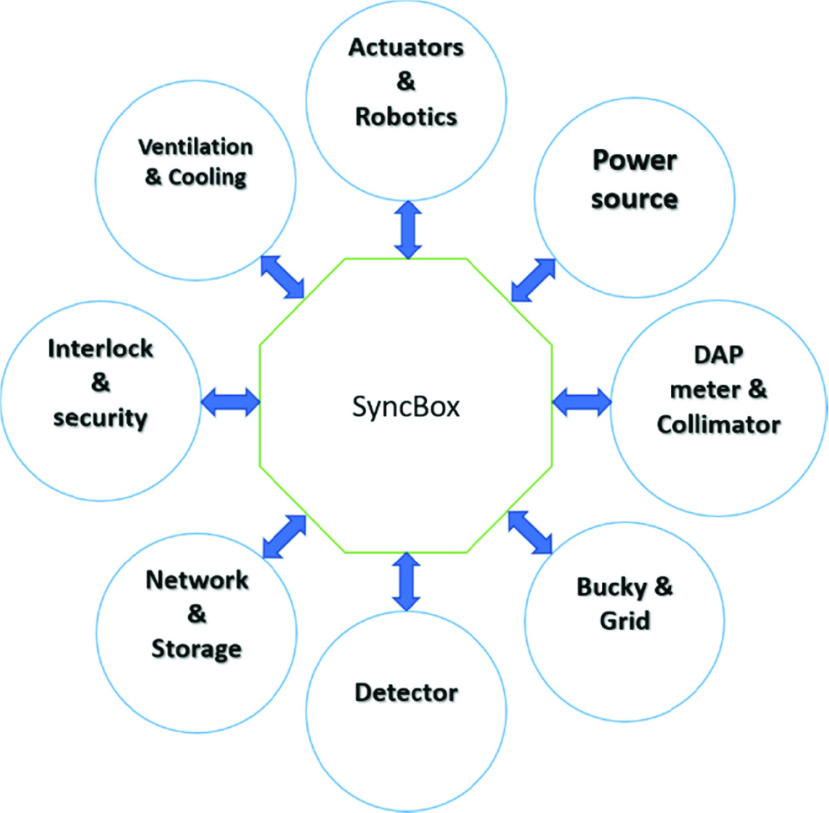
The central hub architecture. SyncBox is placed at the heart of an x-ray scanner. All components are connected to the SyncBox and all communications and transactions are accomplished through it.

A plug-integrate-play (PIP) for x-ray imaging system is introduced here as an open hardware platform; our solution, SyncBox, aims to bring Level-4 interoperability to the radiography devices. SyncBox has designed with central hub architecture (Fig. 2); various compulsory (e.g. detector, high frequency generator, x-ray tube) and additional units (e.g. AEC, robotic movement, DAP, cooling system) utilized in a medical and industrial radiography device (e.g. non-destructive x-ray quality control systems, security x-ray machines) are all connected to this central SyncBox in a star network structure. As a result, all the communications between devices are accomplished through the SyncBox and none of the components is connected to others directly. SyncBox is responsible for providing communication channels between units, translating connection protocols, handling communication standards, managing system-level workflows, message translation and monitoring the overall device security and safety.

Technical and syntactic integrabilities (Level-1 and Level-2 in Table 1) and semantic interoperability (Level 3 in Table 1) for x-ray scanners are mostly related to the controlling and signal handling of the components. Following the ASTM F2761 guidelines, the SyncBox is required to provide isolated and reliable channels between itself and each device. The pragmatic (Level-4) and dynamic (Level-5) interoperability involves more sophisticated problems and usually includes more computationally intensive operations and entangled system workflows between the x-ray scanner and the peripheral devices, such as the archiving systems. Hence, the communications and workflows categorized in various ways: low speed or high-speed data transfer and/or process control. Each category requires different computational capacity and could be implemented differently. At Level-1 up to Level-3, interoperability is achieved via a microcontroller, while Level-4 and Level-5 require a more sophisticated computer such as a mini-computer (also known as Computer on Module - COM).

### A. Hardware Schematics of SyncBox

**FIGURE 3.**
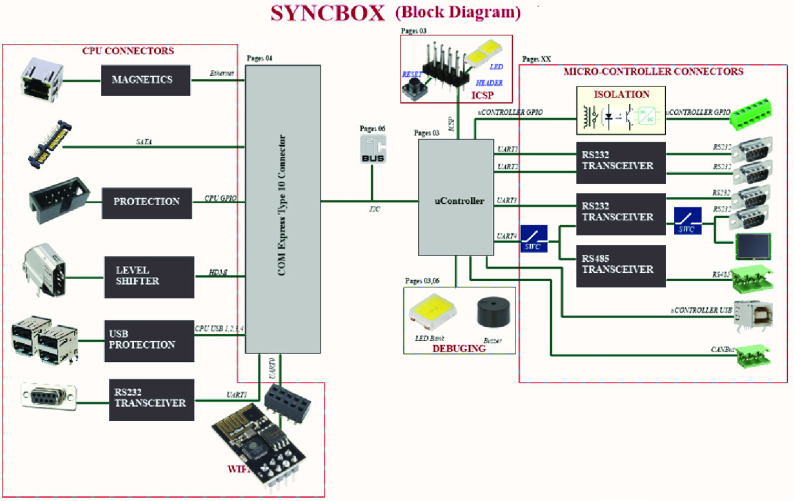
SyncBox Block Diagram with CPU and microcontroller-based connections. Microprocessor Controlled Units and connections are used for advanced operations such as image acquisition, post processing, network-based communication and user interface. Microcontroller Controlled Units and connections are responsible for fast and reliable communications: X-Ray exposure control, emergency and safety sensors, mechanical driver communications.

**FIGURE 4.**
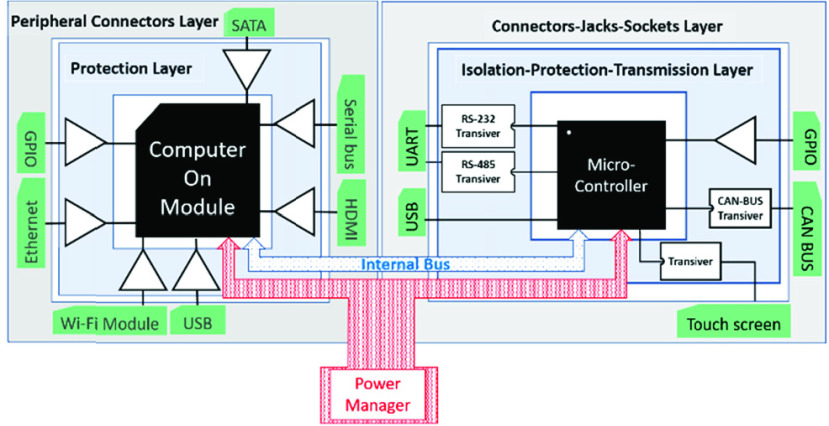
Abstract schematic hardware architecture. Each component of x-ray device (Fig. 2) is connected to one of the peripheral connectors.

SyncBox is a combination of a two-level hardware: a microcontroller-based system and a microprocessor-based platform. Microprocessor controls advanced operations and workflows such as image acquisition, task organization and interacting with users. microcontroller system controls the low-level operations such as device controls, x-ray control and exposure, sensors. SyncBox block diagram showed on Fig. 3 including the CPU and microcontroller-based connectors. The hardware organization schematic of SyncBox implementation is shown at Fig. 4. The Hardware Application Layer (HAL) executes within the microcontroller (Fig. 6).

**FIGURE 5.**
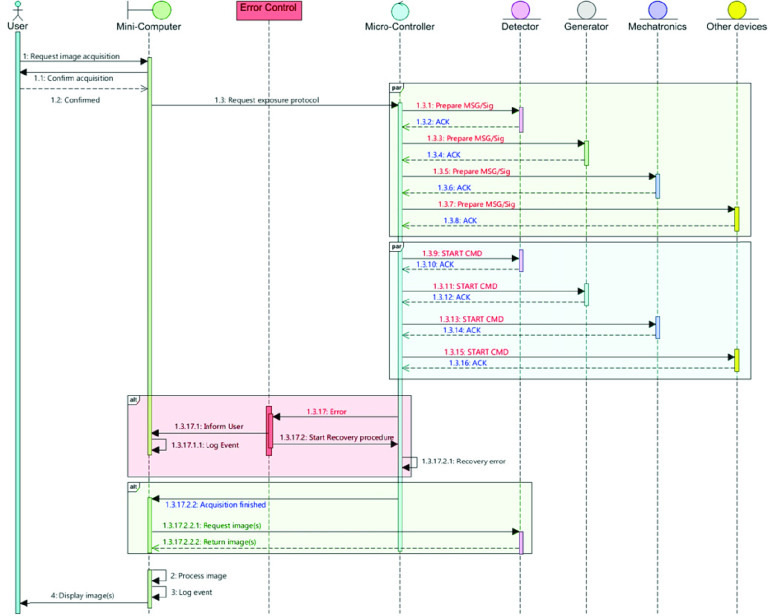
Data model of SyncBox. Transactions are divided into low speed and high speed. Each category is implemented by a special device. A simple work flow diagram for a x-ray protocol.

**FIGURE 6.**
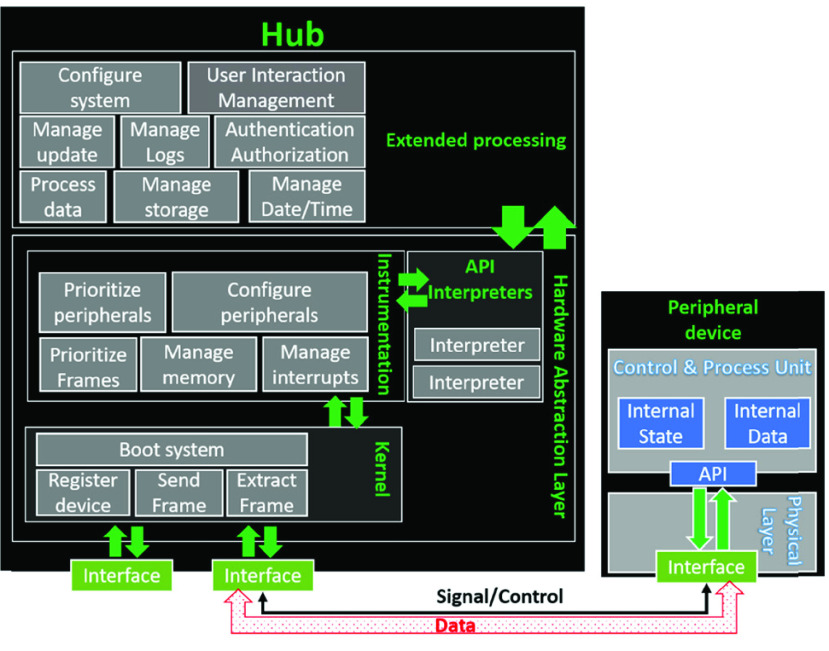
Central hub software architecture. Figure shows the relation and communication between peripheral device and SyncBox software.

The microcontroller and microprocessor are connected to each other over an internal bus and the power manager circuit provides the required powers to all sections, as shown in Fig. 4. The protection layer provides a shielding against ESD and cable discharge event (CDE). The medical standards require circuit protection against electrostatic discharge (ESD), Electromagnetic interference (EMI) and wrong cable plugs. To protect against these disruption sources, all communication headers and junctions are passed through a layer of protection and denoising and then connected to a microcontroller. Some protocols, such as CAN bus and RS-485, also require a special transceiver module. These modules also provide a level of protection against voltage shocks. Finally, the general-purpose input output (GPIO) provides the extension paths for systems with unknown requirements.

### B. Dynamic Model of SyncBox

The dynamic model of SyncBox, as shown in Fig. 6, is applicable to most of the radiography devices, with minor adjustments. A typical workflow is given below.

1) Initially, user defines a set of exposure parameters, such as x-ray energy, intensity and duration to the SyncBox. This interaction between the user and SyncBox is accomplished via SyncBox’s mini-computer or touchscreen display.
2) After getting the user confirmation, SyncBox micro-controller is informed with the imaging tasks. From this step onward, it is the responsibility of the microcontroller unit to handle the interoperability between the devices and orchestrate the ensuring image acquisition.
3) It first prepares all the required devices (such as detector, generator, mechatronics or any other device), then starts the examination.
4) After all previous programmed steps are accomplished successfully, the captured image is grabbed by the mini-computer and displayed to the user otherwise, error recovery mechanism is initiated.

The type and the content of the messages, signals and acknowledgments that is sent to each device is different. Each manufacturer provides a set of instruction guides for their product to describe how to communicate with their components. Besides this, some devices may require communicating with the others directly. In the following section, we will discuss how the SyncBox software and hardware architecture is designed to handle all these requirements.

The extended processing tier (as displayed in the top section of Fig. 6) executes on a computer-on-module express (COMe) unit.

### C. SyncBox Software Architecture Model

SyncBox software architecture is shown in Fig. 5. The hub has a two-tier-architecture: the Hardware Abstraction Layer (HAL), which is executed on microcontroller and the Extended Processing (EP) layer for high-level interoperability tasks run on the mini-computer. The HAL provides the technical, syntactic and semantic interoperation services. EP layer provides a context for data processing and systemic extension ports (Level-4 and Layer-5 in Table 1).

In this architecture, it is assumed that non-trivial peripheral devices (e.g. power generator) have a firmware (or a feedback circuitry) with the internal data and state variables and expose a set of functionalities, known as Application Programming Interface (API), to the outside world. Clients connect to the device through a physical interface and exploit these public APIs. Normally, these API functions accept streams of bits (or bytes) as inputs and produce streams of bits as outputs. However, edge or pulse signals are not uncommon in old devices.

In the core of the HAL, there exists a small piece of code, kernel, which is responsible for fundamental operations. It accepts the messages as a stream of bits, then encapsulate and put them into appropriate memory locations. As a result, it avoids message loss that may occur due to the load of simultaneous flow of incoming messages. One could imagine that the kernel is a virtual secretary that receives a set of unordered papers from different senders, organizes all these incoming papers and places them within appropriate folders [28].

The kernel assigns a specific memory location to each device. These locations are named as logical device slots and hard-coded within the kernel settings Each slot consists of two buffered channels, one for the sent data and the other for the received data, accompanied with a direct channel for non-buffered sent data that is useful for emergency cases. Again, following the same analogy, one could imagine that the secretary assigns three folders for each send-source, one for incoming messages from the sender, the other outgoing messages and the last folder is reserved for top priority messages, which are not actually buffered but directly passed for processing. During the boot process, kernel accomplishes the necessary controls and registers the peripheral devices into their appropriate slots.

Each message stored within the send or receive channel of a slot has a specific format. It consists of a payload header and a delimiter trailer. Delimiter identifies the end of a frame and is usually specified within the peripheral device API-contract. By default, the kernel assumes that the messages are C-Style character strings terminated at a ‘\’, but this assumption is not restricted and it is possible to change the delimiter of each device within the kernel configurations. In order to protect against memory overflow problem, the maximum frame size is set to be constant and determined by MAX_MSG_SIZE kernel parameter.

**FIGURE 7.**
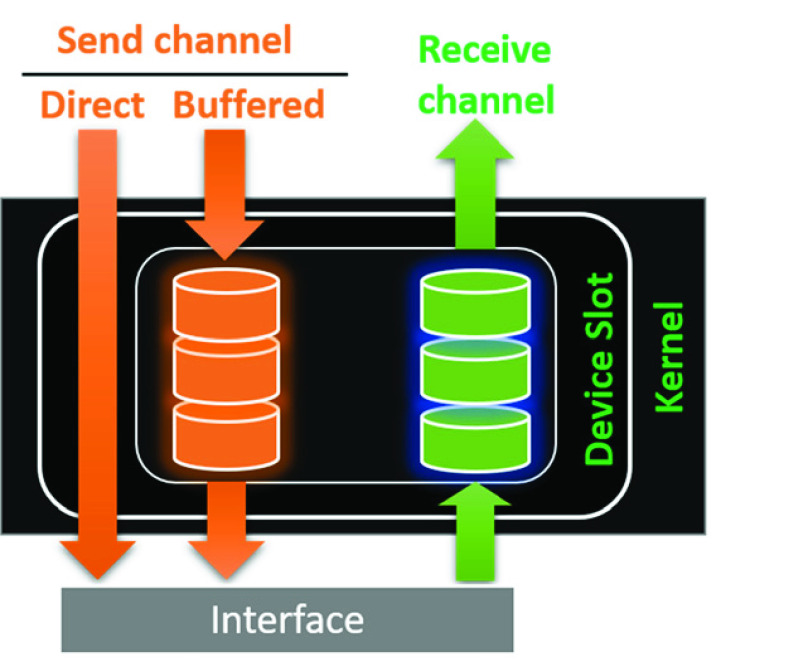
Figure shows a sample device slot structure within the kernel. Each device is a connected to a virtual bus within the kernel. Each slot provides one buffered send channel, one buffered receive channel and one direct pipeline.

Kernel services (Fig. 7) are used by the instrumentation section of HAL. These services relieve the instrumentation from getting involved into communication details (e.g. speed, protocol, control mechanisms, etc.) and allows it to directly receive messages in predetermined and organized frames.

The instrumentation section is called by the kernel after a successful boot-up. The kernel orchestrates the system workflow, interprets the incoming interrupts and triggers the peripherals with the most updated situations. The peripheral devices do not carry the same weight of importance. Some devices are required to respond within a specific time interval, otherwise malfunctions could occur. Most common malfunction scenarios are asynchronous work between x-ray and detector which results with insufficient or empty imaging result, delay or failure on the collision sensors. As a more serious example, if actuators do not receive a stop command in a timely manner, the patient may be harmed. In addition, messages of the same device type may have different levels of priorities. The instrumentation design is responsible for both types of prioritizations (i.e., prioritizing devices and messages). Instrumentation system is the core control section of the system. SyncBox device works with several peripheral devices by using their application programming interface (API). SyncBox main task is to monitor the actions coming from API’s, prioritize them and send them to necessary peripheral devices in order. Instrumentation section requires to interact with peripheral APIs. Each manufacturer provides its own set of API and there is no common agreement on the details of the public functions signature. In order to alleviate this issue, the instrumentation employs interpreters. For each device type of each vendor, an interpreter is required for the instrumentation. Interpreters translate the messages coming from the peripheral API into a standard language that the instrumentation could understand. In the ideal case, whenever a peripheral device is replaced, it should be enough to add the appropriate interpreter to the interpretation section. In addition, a device manufacturer may revisit the instrumentation section for optimizing their own setup if the default workflow does not meet their requirements.

### D. Extended Processing

The Extended Processing section is a collection of bulky operations, such as handling user interface, protocol preparation (patient type, energy level, mechanics positioning parameters), image processing, data management and integration with Radiology Information System (RIS) and Picture Archiving and Communication System (PACS). These operations are totally realizable in a high-level software (such as C++ or C#) and to execute such a program, an operating system is required (e.g. Linux, Windows).

## IV. Results

**FIGURE 8.**
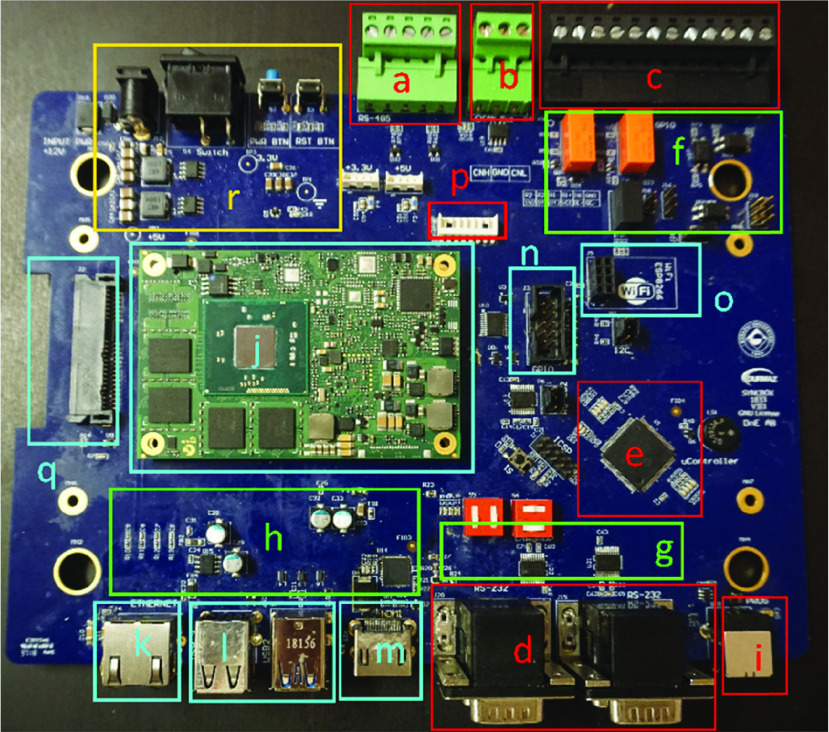
Implemented SyncBox device. The details of each unit are listed in Table 2. x-ray systems could use a variety of communication protocols. SyncBox has all the generic communication ports ready for future applications.

First hardware implementation of the SyncBox is shown in Fig. 8, where each labeled section (A-R) presented is described in detail at Table 2. Both hardware design and the developed firmware are freely available [29] community members could download all the associated results and documentations and rebuild the work, or it could be customized for similar devices.

**TABLE 2:** Units for Magnetic Properties SyncBox Sections. Table Shows All Main Sections Included in SyncBox. I/O Ports, Power Circuit Design, a Microcontroller, a Micro pc, Display and Storage Units

Section	Title	Details
a	RS-485	Full Duplex Protocol
$b$	CAN bus	Three Pins Header
$c$	GPIO	Single and Paired GPIO
$d$	RS232	No Parity, no handshaking 8-bit
$e$	PIC24EP512GU810	16-bit microcontroller
f	Isolation and Protection	Input direction, output direction and Input-Output direction
g	Transceiver	Serial bus connectors
$h$	Transceiver	High speed connectors
$i$	In-circuit serial programming (ICSP)	Upgrade microcontroller firmware
j	COMe Type 10 mini	Intel Celeron with 4GB RAM
k	Ethernet	Gigabit ethernet
l	USB II and III	Connected to COMe
m	HDMI	COMe display out
$n$	GPIO	Connected to COMe
$o$	Wi-Fi socket	ESP8266 Wi-Fi Module
$p$	Touch screen socket	Microcontroller connector
$q$	SATA	External hard disk
$r$	Power Manager	12V input to +5V and +3.3 V

**TABLE 3:** Comparison Between 3 Whole Body x-Ray Scanner According to Literature Findings. Resolution and Pixel Pitch Information is Not Available for Eos System

	LODOX/ Statscan	EOS	X-Lab TDI based scanner
Scanning Time (seconds)	13	20	18
Radiation Dose (mGY)	0,33	0,92	0,76
Pixel Pitch ($\mu \text{m}$)	60	N/A	27
Image Resolution (lp/mm)	5	N/A	9
Main Focus	Trauma / Fractures	Scoliosis	Trauma / Emergency
Approximate Price	$ 335.000		$200.000

The firmware was prepared with MicroC pro version 7. The floating-point numbers were based on IEEE 754 32-bit and all integer values were two bytes little endian. The microcontroller is externally tuned with an 8-Mhz crystal that is internally raised up to 140 Mhz.

**FIGURE 9.**
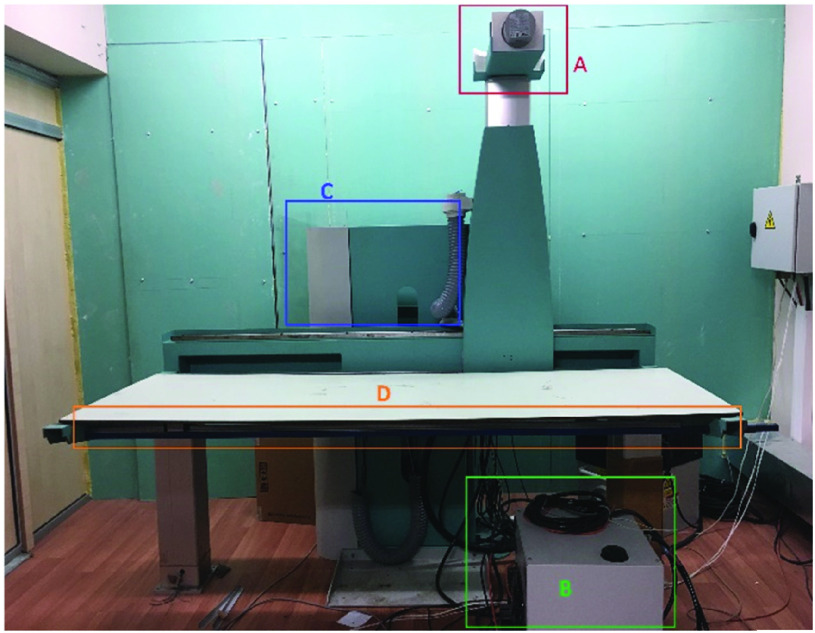
A TDI line scanner based full body x-ray scanner. SyncBox was employed to design and operate this configuration. A) X-ray tube B) High frequency generator system, C) Main body with servo motors, controllers and electrical system, D) Patient table combined with detector system. x-ray system includes all the main and some additional component that would be present in a standard x-ray system. For minimizing shifting between source, and detector, x-ray tube and line detector are moving along the same mechanical unit controlled by a single motor.

The proposed architecture was implemented and used for assembling a full body scanner (Fig. 9). In this device, Teledyne Time Delay Integration (TDI) detector (located under the patient table, labelled as Fig. 9-D), Gulmay High frequency generator (B), x-ray tube (A) and Delta B2 motor controllers (C) were used. TDI (Time Delay and Integration) Sensors idea firstly developed during 1970’s. A multiline of CCD detectors sum up the intensity’s during the procedure. By new technologic developments covering the TDI sensors with fiber optic, and CsI (Cesium Iodine) scintillator surface X-Ray directly converts into visible photons and captured by the TDI camera. Main advantage of the TDI sensor is the speed and high resolution, regarding to other digital x-ray systems [30]. TDI has sensors are used for metal NDT, historical object scanning, medical, dental and document analysis [31], [32] TDI has an advantage for fast scanning time, and high resolution on our application.

The developed system was designed with moving detector-source and static-object during x-ray exposure. TDI detector technology has a very high scanning resolution, but they are very sensitive to mechanical vibrations. In our device, the detector could scan 166 lines per cm and each line has approximately $27~\mu \text{m}$ resolution. To satisfy these requirements, the robotic unit was required to provide precise movement and stop mechanisms on a very short vibration amplitude. The robotic system could offer $20~\mu \text{m}$ step accuracy, up to 10 cm/sec movement speed. The details of the device connections are listed in Table 4.

**TABLE 4:** Units for Magnetic Properties Full x-Ray Scanner Connections. This Table Shows an Example x-Ray System Main Component Communication Protocols. These Protocols are the Common Standards for x-Ray Devices

Device	Type	Protocol	Connection
TDI - Teledyne Argus	Detector	Ethernet	GigE-RJ45
Servo Motor Driver −− Delta B2	Actuator and Robotics	Modbus RTU	RS-232-dsub9
Power Generator Gulmay JA3200	Power Source	UART	RS-232-dsub9

A comparison has been made between commercial whole body imaging devices on the market. LODOX / Statscan is a digital x-ray scanner [33], [34]. It is mostly specialized in trauma patients, and fracture detection. EOS is a European based system, using a gaseous detector named MicroMegas [35] by G. Charpak et.al. TDI based whole body scanner developed in X-Lab, Boğaziçi University for to provide the proof of concept about our controller platform SyncBox. Conventional stitching method, Lodox, EOS, our new design. Table 3 is a basic comparison between 3 systems based on literature and technical findings.

The device was tested for both communication diversity and image consistency in order to show that data flow was provided without loss and error.

### A. Servo Driver Test

**FIGURE 10.**
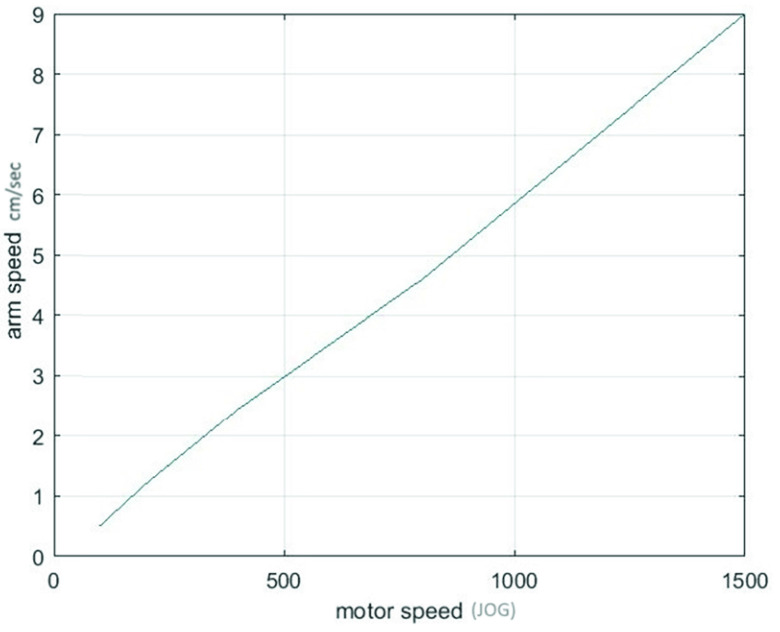
Motor speed connection chart. Experimental results of SyncBox communication with servo motor drives. The graph indicates the linearity between speed and control JOG parameter input. It is used to observe the stability of mechatronic system control of the SyncBox system.

The servo driver was tested for measuring the smoothness of the arm movement. A range of motor rotation speeds was sent to the Delta-B2 servo controllers and the actuators move was measured (Fig. 10). The output clearly depicted a smooth and linear relationship between the requested speed and the output displacement.

### B. SyncBox Adaptability - Detector Switching

**FIGURE 11.**
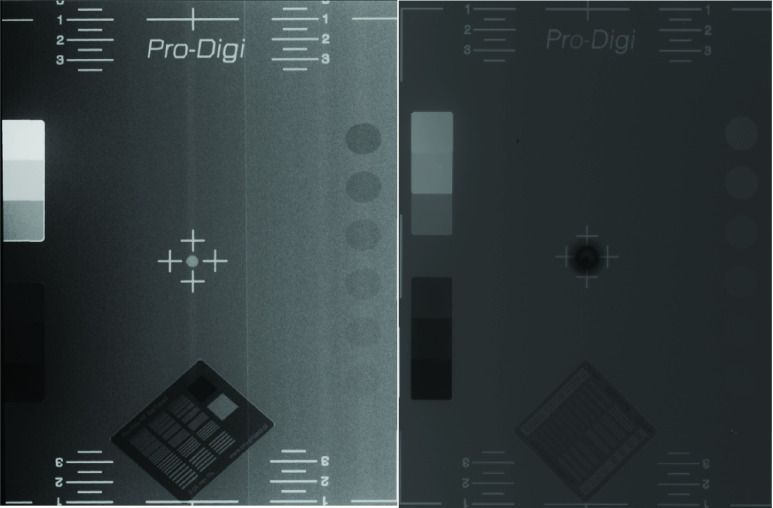
Pro-Digi Pro-Project −02 -102 phantom images for (left) a TDI detector and (right) a flat panel detector. Phantom images have been taken with same x-ray tube and generator. 8:1 ratio, 215 line / inch aluminum grid has been used for flat panel detector. Results shows that TDI scanner has higher performance on contrast and resolution comparing to flat panel.

**FIGURE 12.**
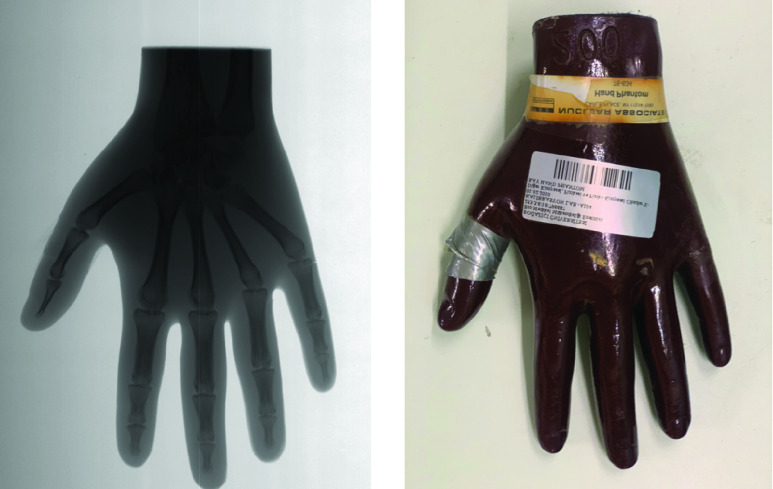
Victoreen Nuclear Associate Hand Phantom 76–634 x-ray image taken by SyncBox controlled TDI detector x-ray scanner. 0.5 mm aluminum filtration has been used. Exposure parameters are 60 kVp, 10 mA duration 1.8 sec. Total image acquisition time 5 seconds.

SyncBox unit was tested with two different sensors in order to prove that it is adaptable to be used with different components on an x-ray device. For this purpose, we changed the current TDI detector with a Toshiba FDX4343R CMOS Flat panel detector. We used Pro-Digi Pro-Project – 02-102, a CE certified phantom, which complies with IEC 61223–3-1 and DIN 6868/58 standards. Fig. 11 shows x-ray images taken with two different detectors using the same generator and x-ray tube system along with the SyncBox interface. During this process, one detector was taken out of the system, while the new detector was installed with its own bucky unit. We have used the same generator and x-ray tube for testing. We have widened the collimator setting for the flat panel to able to get full imaging of the phantom. Mechanic movement system was disabled during flat panel image acquisition. The performance of images compared by using ImageJ histogram tool. Each layer of step wedge phantom inside Pro-Digi Pro-Project – 02-102 has been checked for histogram. Mean contrast values and standard deviation values are recorded for homogeneity and contrast resolution [36]. According to results in Table 5 TDI detector offers higher contrast. Fig. 12 shows x-ray image using an anthropomorphic hand phantom under 2 seconds.

**TABLE 5:** Detector Contrast Comparison by Using Step Wedge Phantom Inside Pro-Digi Pro-Project -02 -102. Each Step Histogram has Been Calculated Using ImageJ. Comparison Variables are Mean Contrast Value and Standard Deviation

Step Value	TDI Detector		Flat Panel Detector	
	Standard Deviation	Mean Contrast Value	Standard Deviation	Mean Contrast Value
0	3.196	247	3.191	116
1	4.746	155	1.744	108
2	2.148	103	1.180	85
3	1.147	69	1.125	62
4	0.694	47	0.977	43
5	0.633	37	0.809	32
6	0.710	32	0.982	26

## V. Discussion

The advancement in interoperable devices for medical applications is relatively new all through for the last 15 years. Academicians and regulatory bodies are actively working to find applicable solutions for these demands [37]–[39]. Open source platforms that supply physical infrastructure for data and signal transfer between medical systems are emerging; these allow devices and applications to be connected or disconnected on demand [40]. The standards, developed by regulatory bodies, sketch a generic system model for device interoperation, the equipment safety, security and usability characteristics. However, absence of a serious open source effort could meet these criteria impede the progress of the available platforms [24], [41].

Successful integrability in medical devices could provide with open source and Plug-and-Play (PnP) streamlines to meet researchers’ extensibility demands. Open source movement provided stable, elegant and low cost alternatives in daily life applications [36] and the PnP paradigm has a potential to ease device interoperability and human-machine interactions. There are several examples of medical open source hardware solutions in literature such as electrocardiogram, myoelectric prostheses, infusion pumps, physiological monitoring, EEG systems and even a CT Scanner [40], [43]. However, due to the tight regulations of medical device ecosystem, the users does not prefer to use open source solutions and PnP systems in order to comply with interoperable medical device standards [16], [20]. Recently, there have been some attempts to change this mindset and bring the medical device industry closer to the PnP paradigm and make this industry more attractive for the open source community [20].

In practice, Medical PnP has already successfully been applied for sharing electronic health information [26]. A group at the University of Florida prototyped a system to synchronize x-ray radiography device with the patient’s breathing cycle to improve the quality of the radiography on a ventilated patient [13]. There are other examples, where medical device interoperability and medical system integration improved patient safety especially on clinical engineering area such as prioritizing ventilation system alarms [10], [20]. Unfortunately, due to the inability to cross the “interoperability chasm”, the slow pace medical device and health information technology (HIT) ecosystem is deprived from many other good ideas for improving patient care [10], [44].

In this paper, a new open source platform for developing interoperable radiography devices is introduced, a version of this platform is implemented as SyncBox and is applied for developing a full body x-ray radiography device. Despite the available alternatives, the SyncBox targets to reuse existing components and infrastructure to extend the creativity with modular blocks for developing safe and interoperable radiography systems. The actuator relocation smoothness and image quality were evaluated and the results showed that both criteria were satisfied within acceptable intervals. Additionally, a TDI detector was replaced by a Flat panel technology detector and SyncBox successfully integrated the detector with minimum efforts.

This platform aims to accelerate the realization of innovative novel designs with potential gain in development costs and time. This work is timely, since there is a trend within the radiography systems, shifting from “as-is” to “plug-integrate-play (PIP)” as the hardware design principle.

In contrast to the other open source interoperability platforms [40], [45], [46], which aim toward making connection in between end systems, the SyncBox brings the concept of the interoperability to the device design phase. The manufacturers and developers could build up a system specifically to meet their expectations using SyncBox. Health institutes, academic researchers and regulatory bodies could assemble their own customized radiography scanners based on their own necessities. This platform democratizes the x-ray device manufacturing process and breaks the prefabricated device design limitations (such as detector and power source choices) on acquired data. In long term, this platform could lead the way for more cost-effective radiography devices and reduce the device manufacturing time.

SyncBox has a potential to extend its usage area to many platforms, starting with fluoroscopy and mammography devices due to working principle to direct x-ray systems. In theory it could be extended in the future to support other modalities, such as ultrasound devices or multimodality imaging systems. It has a potential to communicate and control many different devices which could be extended to several other hardware-like transducers, or advanced mechanics. However, these implementations need a collaborative effort and significant support from different imaging device component vendors and research teams, for the required API integrations and software development.

Performance comparison between SyncBox and conventional OEM system development has been made using several factors (Table 6). SyncBox offers flexible solutions for end users, on the other commercial products may offer more stable products. SyncBox offered as an open source platform for users, however device cost is depending on several variables such as component prices and integration cost. SyncBox has several advantages over the OEM and pre designed systems on integrability, extensibility, upgradability, customizability.

**TABLE 6:** SyncBox Based – OEM Device Comparison. SyncBox Based Device Could be Upgrade, Customized or Convert to a Different Modality Such as From DR to a Mammography. Device Components Could be Replaced Easily. on the Other Hand, There Could be Problems on the Stability, and Certification Process Such as CE or FDA Should be Completed by the End User

Section	SyncBox based X-Ray System	OEM X-Ray DR Systems
Integrability	Full	Partial
Extensibility	Full	None
Upgradability	Full	Partial
Customizability	Full	Partial
Standard Compatibility	Partial	Partial
Inter-operability	Full	Partial
Stability	Relative	Stable
Cost	$50.000 - $400.000	$80.000-$300.000

For future, we aim to improve our design and build up additional SyncBox based x-ray imaging modalities, such as fluoroscopy and mammography. If the necessary regulatory approvals could be obtained, preferably with the assistance of one of the device production companies, we plan to complete the clinical testing.

## VI. Conclusion

The SyncBox model and its implementation followed the best practices of software and electrical engineering. All aspects of the system were modeled and documented in different levels of technical details. These models and documentations are shared across communities of researchers and manufacturers to enable them to leverage and reuse, with their own software and hardware components.

We believe that the “SyncBox approach” has a great potential for cost effective, standardized, and faster prototype production for innovative medical imaging devices. Once the SyncBox is applied by different research groups and improved further within its open platform format by all, it could have a chance in becoming a standard R&D tool for innovative medical imaging products in the future.
